# Does size of the cochlear nerve affect postoperative auditory performance in pediatric cochlear implant patients with normal cochlear nerves?

**DOI:** 10.1016/j.bjorl.2020.06.019

**Published:** 2020-08-08

**Authors:** Emine Deniz Gozen, H. Murat Yener, Halide Kara, Ahmet Atas, Osman Kizilkilic, Harun Cansiz

**Affiliations:** a Istanbul University Cerrahpasa Medical Faculty, Otorhinolaryngology Department, Istanbul, Turkey; b Istanbul University, Faculty of Health Science, Audiology Department, Istanbul, Turkey; c Istanbul University Cerrahpasa Medical Faculty, Radiodiagnostic Department, Istanbul, Turkey

**Keywords:** Cochlear implantation, Prelingual deafness, Cochlear nerve morphology, Auditory perception

## Abstract

**Introduction:**

Cochlear implantation is an effective treatment method for severe to profound hearing loss. Many factors that may influence cochlear implantation success have been explained in previous studies. Apart from those, minor differences in size of normal cochlear nerves may affect postoperative performance.

**Objective:**

To investigate whether the minor differences in cochlear nerve size in normal cochlear nerves affect postoperative cochlear implant performance.

**Methods:**

30 pediatric prelingually deaf patients who were treated with cochlear implantation were included in this study. From the reconstructed parasagittal magnetic resonance images, the diameter and cross-sectional area of the cochlear nerve on the ipsilateral and contralateral side were measured. Auditory evaluations were performed 1, 3, 6 and 12 months following the first fitting. All the analysis was performed by using EARS®, evaluation of auditory responses to speech tool. Correlation between cochlear nerve diameter, cross-sectional area and postoperative auditory perception was analyzed to determine whether variation in cochlear nerve size contributes to postoperative auditory performance.

**Results:**

The mean diameter of the cochlear nerve on the ipsilateral side was 718.4 μm (504.5 − 904.3 μm) and mean cross sectional area was 0.015 cm^2^ (0.012 − 0.018 cm^2^). On the contralateral side the mean cochlear nerve diameter was 714.4 μm (502.6 − 951.4 μm) and mean cross sectional area was 0.014 cm^2^ (0.011 − 0.019 cm^2^). The correlation between the diameter and cross-sectional area of the ipsilateral and contralateral cochlear nerve revealed no significance. Mean score at first month monosyllable-trochee-polysyllable test, MTP1, was 0.17 (0.08 − 0.33), at 6th month with 6 words test, 6th month MTP6 was 0.72 (0.39 − 1.0), at 6th month with 12 words, 6th month MTP 12 was 0.46 (0.17 − 0.75) and at 12th month with 12 words, 12th month MTP12 was 0.73 (0.25 − 1.0). There was no correlation between the monosyllable-trochee-polysyllable test, values at any time with the diameter of the ipsilateral cochlear nerve. However, the first month MTP, 6th month MTP6 and 12th month MTP12 correlated with the cross-sectional area of the ipsilateral cochlear nerve.

**Conclusion:**

Measuring the cross sectional area of the normal- appearing cochlear nerve may give important prognostic knowledge on cochlear implant outcomes. In patients with a larger cross sectional area the auditory performance was better and faster. Although normal appearing, slight differences on cross sectional area of the cochlear nerve may affect performance. Measuring the size of the cochlear nerve on parasagittal magnetic resonance images may provide beneficial information on the postoperative rehabilitation process.

## Introduction

Cochlear implantation is an effective treatment method for those with severe to profound hearing loss by introducing signals directly to the neurons in the spiral ganglion, which transfers the impulses to the cochlear nerve. The main expected outcome of cochlear implantation is to achieve normal or near- normal speech and language skills in prelingual deaf children and to achieve normal hearing in post- lingual deaf individuals. But the expected outcomes may differ according to individual factors and pos- implantation training and education.^1^, ^2^, ^3^

Many factors that may influence cochlear implantation success have been explained in previous studies. Among these, presence of additional disabilities^4^ parental influences, socioeconomic status, involvement of the caregivers to education course, maternal education,^5^, ^6^, ^7^ prematurity, low birth weight, intensive care unit admission and intubation^8^ are all described. Black et al. studied different variables such as age at implantation, family, additional problems, complications during surgery, gender, GJB2 mutation, meningitis, malformations of the inner ear and prematurity in their review manuscript and found that the only significant prognostic factors were inner ear malformations, influence of the family and late implantation.^9^ In general patients with early intervention and prior use of hearing aids are considered to have better prognosis.^10^, ^11^

As there is no accurate method for determining the outcome of cochlear implants, the patients should be evaluated in detail for candidacy. Cochlear malformations, cochlear nerve abnormalities and ossification of the cochlea are well-studied disorders that may increase the likelihood of poor prognosis due to highly variable results.^12^, ^13^ One of the most challenging cases among these malformations is cochlear nerve abnormality. High resolution MRI has been traditionally used for identification of cochlear nerve abnormalities. The diameter of the cochlear nerve was evaluated in several studies. Morita et al. measured cochlear nerve diameters from axial magnetic resonance images (MRI) in cochlear implant candidates and correlated it to postoperative outcomes and demonstrated that patients with larger nerves performed better.^14^ Measurements from parasagittal MRI were performed in different studies, which yielded more accurate findings.^15^, ^16^ Sildiroglu et al. calculated cross-sectional area of the cochlear nerve in elderly patients with sensorineural hearing loss and could not find significant changes in the cochlear nerve size.^17^ Russo et al. found mild hypoplasia of the cochlear nerve in children with profound hearing loss on MRI.^18^ In a more recent study Chung et al. detected that patients with either deficient cochlear nerve or a narrow bony cochlear nerve canal had poor outcomes after cochlear implantation.^19^ The facial nerve has been previously used to assess the cochlear nerve size comparatively, and as a quick reference the facial nerve to cochlear nerve ratio can be used to evaluate cochlear nerve anomalies.^20^ However, there is limited data on the effect of minor differences in cochlear nerve size in non-hypoplastic or deficient cochlear nerves on postoperative cochlear implant performance. In one study in which cochlear implantation was performed on post- lingually deaf patients, the cross-sectional area of the cochlear nerve was positively correlated to auditory performance.^21^ Even if the cochlear nerve is hypoplastic, the patients can benefit from cochlear implants. The presence of the cochlear nerve on MRI and no other anomalies accompanying hearing loss were found to be associated with better hearing outcomes.^22^

In this study we therefore aimed to measure the diameter and the cross-sectional area of the normal- appearing cochlear nerves in patients who received cochlear implants and analyze the relation between cochlear nerve size and postoperative performance that may help to predict cochlear implant outcomes.

## Methods

### Ethical considerations

The study was performed in otolaryngology department following approval by the institutional review board (approval number: 279851). Informed consents were retrieved from the parents of all the patients. Patient data and images were stored in the electronic patient records and analyzed retrospectively.

### Patients

Medical records of 180 prelingual bilateral deaf pediatric patients who received cochlear implants between years 2011 − 2015 were reviewed retrospectively for otolaryngologic examinations, preoperative hearing tests, temporal bone computed tomography (CT) and MRI. All of the patients had congenital hearing loss and in the preoperative auditory brainstem response (ABR) examination none of the patients had bilateral wave 5 at 90 dB nHL. Patients with less than one year followup, incomplete medical records, unavailable imaging, inner ear anomaly, incomplete electrode insertion, and postoperative complication were excluded. After exclusions, a total of 30 patients were included in the study that had non-hypoplastic or aplastic cochlear nerves in the MRI when compared to facial nerve. Twenty of the patients were male and 10 of them were female with ages at the time of implantation ranging between 11–56 months with a mean of 19.2 months.

All of our patients were tested by electrophysiologic and behavioral methods. All subjects were first tested with otoacoustic emissions (DPOAE), followed by chirp and narrow band chirp stimulus and ABR tests. Cochlear implants were applied to patients whose DPOAE could not be obtained and whose threshold values were not obtained in the intensity over 90 dB nHL in ABR test (no threshold was obtained in bone ABR). Behavioral tests were performed to patients who can cooperate during the tests, to determine the hearing thresholds by behavioral observation vudiometry (BOA) or Visual reinforcement audiometry (VRA) of play audiometry. Cochlear implant decision was made for patients who did not demonstrate threshold over 90 dB in 500 − 4000 Hz range in the behavioral tests. According to the audiologic findings and the dominant hand use of the patients the ears to be operated were selected.

After comprehensive preoperative hearing evaluation and temporal bone CT and MRI images, cochlear implantation decision was made and under general anesthesia standard cochlear implantation by mastoidectomy and round window insertion was carried out. Most of the patients received right ear implantation (n = 29, 96.6%). The cochlear implants were Nucleus™ in 11 patients, Medel™ in 11 patients and Clarion™ in 8 patients.

### Imaging and measurement of cochlear nerve

All patients underwent temporal bone CT and MRI in the Neuroradiology Department. Conventional brain and inner ear scanning were performed using a 3 T MR scanner. High-resolution 3-D constructive images in steady state (CISS) − sequence MRI of the temporal bone was acquired. In this sequence, data was acquired with a field of view of 160 mm, slice thickness of 1.5 mm, and an imaging matrix of 192 × 512. The CISS- sequence images were transferred to Macintosh Osirix™ software for analysis. Following the identification of internal acoustic canal in axial images, parasagittal reformatted images were created by 3D Multiplanar Reformatting mode (3D MPR) of the OsiriX program. The reformatted images were perpendicular to the long axis of the Internal Acoustic Canal (IAC) from the exit point of cochlear nerve to the cochlea which demonstrated the real axial images of the nerve. From the axial view sagittal and coronal planes were moved over the IAC and images were rotated so that the axis of rotation was perpendicular to the IAC. The calculations were performed in the midpoint of the cochlear nerve from the exit point from cochlea and entry point to the brain stem, both on the ipsilateral and contralateral side. The horizontal diameters of the cochlear nerve were measured on the reformatted parasagittal images and were used to calculate cross sectional area of the nerve by two different radiologists separately and the mean value of the different measurements were used.

### Evaluation of auditory outcome

Auditory evaluations were performed 1, 3, 6 and 12 months following first fitting. All the analysis was performed by using EARS® (evaluation of auditory responses to speech) tool which was initiated by Dianne J. Allum-Mecklenburg in 1995.^23^ The assessment tool contains several speech tests and questionnaires and has been adapted to more than 20 languages since its first presentation in 1996. This tool is suitable for children aged 1–18 years. Form this test tool Turkish version of monosyllable-trochee-polysyllable test (MTP) with recognition of one, two or three syllable words with 6 or 12 words (MTP6 and MTP12 respectively) and Monosyllable Closed-Set test performed by bisyllable words (BIS12) were used.

### Monosyllable-Trochee-Polysyllable test (MTP)

Following the fitting the patients were evaluated at 1st, 6th and 12th months. Tests with 3, 6 and 12 pictures were applied at the first month, 6th month and 12th month, respectively. Each word was repeated and evaluated 4 times in 3-word test (total 12 points), 3 times in 6-word test (total 18 points) and 2 times in 12-word test (total 24 points). For statistical analysis the points were converted to percent values.^24^, ^25^ Also the patients were evaluated as pass or fail according to 70% success rate in each test.

### Monosyllable closed-set test performed by bisyllable words (BIS12)

This test that was first used by Schneider et al.^26^ was done with 12 pictures with bisyllable words and evaluated over 24 points. The points were then converted to percentage values for statistical analysis.

### Statistical analysis

The recorded data of cochlear nerve diameter and cross-sectional area were imported into the SPSS 22.0 statistical package (SPSS, Inc., Chicago, IL). The Kolmogorov-Smirnov test was used to compare the distributions of the data to the normal distribution. For the analysis of quantitative data the Mann-Whitney *U* test was used. To analyze repetitive measurements the Wilcoxon test was performed. The Spearman correlation was calculated to determine the correlation between cochear nerve (CN) diameter, cross-sectional area, and other parameters. A *p*-value < 0.05 was considered statistically significant.

## Results

**Figure 1:**
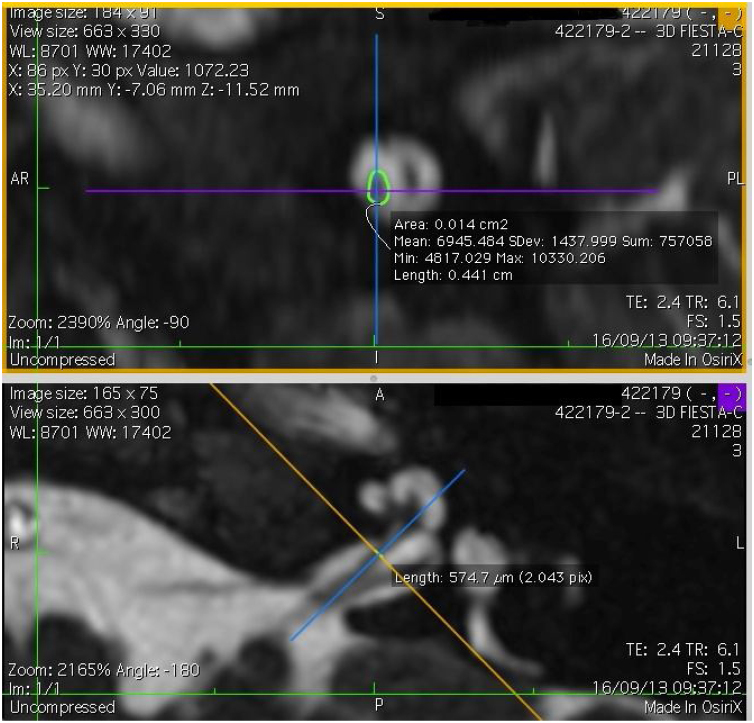
3D Multi-Planar Reformatting mode (3D MPR) in OsiriX site of the calculated diameter of the cochlear nerve.

**Figure 2:**
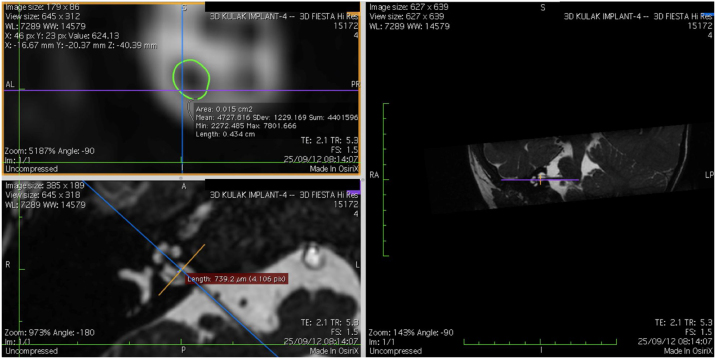
3D Multi-Planar Reformatting mode (3D MPR) in OsiriX site of the calculated diameter of the cochlear nerve.

**Figure 3:**
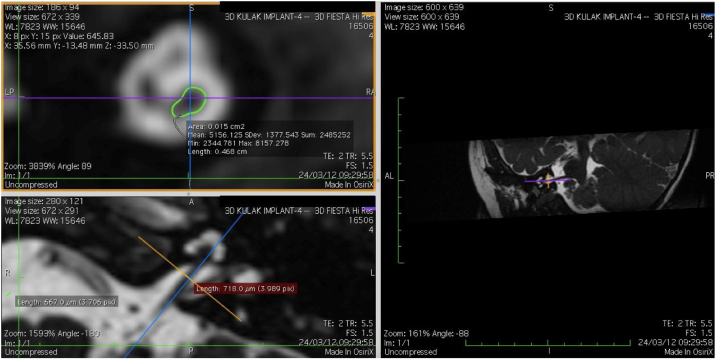
3D Multi-Planar Reformatting mode (3D MPR) in OsiriX site of the calculated diameter of the cochlear nerve.

The cochlear nerves of 60 ears of 30 patients were measured using reformatted parasagittal images in OsiriX software. Figures show the reformatted image and the site of the calculated diameter of the cochlear nerve (Figure 1, Figure 2 and 3). The demographics, mean, median and standard deviation of cochlear nerve measurements from ipsilateral and contralateral sides and EARS test results are presented in Table 1.

**Table 1:** The demographics, mean, median and standard deviation of cochlear nerve measurements from ipsilateral and contralateral sides and EARS test results.

		Min − Max	Median	Mean ± SD, n (%)	
Implantation age (month)		11.00 − 56.00	16	19.10 ± 9.10	
Gender	Female			10	0.33
	Male			20	0.67
Implantation side	Right			29	0.97
	Left			1	0.03
1^st^ month MTP		0.08 − 0.33	0.17	0.17 ± 0.09	
6^th^	Fail			14	0.47
	Pass			16	0.53
6^th^ month MTP6		0.39 − 1.00	0.67	0.72 ± 0.16	
6^th^ month MTP12		0.17 − 0.75	0.38	0.46 ± 0.17	
12^th^	Fail			5	0.17
	Pass			25	0.83
12^th^ month MTP12		0.25 − 1.00	0.75	0.73 ± 0.19	
12^th^ month BIS12		0.13 − 0.92	0.58	0.57 ± 0.23	
Cochlear nerve					
İpsilateral diameter (µm)		504.50 − 904.30	718.4	699.75 ± 106.71	
Contralateral diameter (µm)		502.60 − 951.40	714.4	704.30 ± 114.18	
İpsilateral area (cm^2^)		0.012 − 0.018	0.015	0.015 ± 0.001	
Contralateral area (cm^2^)		0.011 − 0.019	0.015	0.014 ± 0.002	

When the diameter of the ipsilateral side was compared to the contralateral cochlear nerve diameter there was no significant difference between the measurements. The mean diameter of the cochlear nerve on the ipsilateral side was 699.75 ± 106.71 μm (504.5 − 904.3 μm) and mean cross sectional area was 0.015 cm^2^ (0.012 − 0.018 cm^2^). On the contralateral side the mean cochlear nerve diameter was 704.30 ± 114.18 μm (502.6 − 951.4 μm) and mean CSA was 0.014 ± 0.002 cm^2^ (0.011 − 0.019 cm^2^) (Table 2). The correlation between the diameter and cross-sectional area of the ipsilateral and contralateral cochlear nerve and the age at the time of implantation revealed no significance (*p* > 0.05) (diameter ipsilateral and contralateral vs age: *p* = 0.55 and *p* = 0.98 respectively, r = 0.13 and r = 0.003 respectively).

**Table 2:** İpsilateral and contralateral diameter and cross-sectional area of cochlear nerves.

Cochlear nerve	Min − Max	Median	Mean ± SD	*p*
Ipsilateral diameter (µm)	504.50 − 904.30	718.4	699.75 ± 106.71	0.79
Contralateral diameter (µm)	502.60 − 951.40	714.4	704.30 ± 114.18	
Ipsilateral area (cm^2^)	0.012 − 0.018	0.015	0.015 ± 0.001	0.36
Contralateral area (cm^2^)	0.011 − 0.019	0.015	0.014 ± 0.002	

Wilcoxon test.

In the MTP tests with one, two and three syllable words on 1st, third, 6th and 12th months following fitting the findings were as follows: mean score at first month MTP (MTP1) was 0.17 (0.08 − 0.33), at 6th month with 6 words test (6th month MTP6) was 0.72 (0.39–1.0), at 6th month with 12 words (6th month MTP 12) was 0.46 (0.17−0.75) and at 12th month with 12 words (12th month MTP12) was 0.73 (0.25 − 1.0) (Table 3). The correlations among MTP values were significant between MTP1, MTP6, MTP12 at 6th month and MTP 12 at 12th month (*p* = 0, 0, 0.003, r = 0.765, 0.78, 0.608 respectively), 6th month MTP12, 6th month MTP12 and 12th month MTP12 (*p* = 0 and 0, r = 0.989 and 0.779 respectively (Table 3). The correlation analysis also detected that although not significant, MTP values were correlated reversely with the age at implantation (better the MTP smaller the age at implantation) (age at implantation vs MTP1, MTP6, 6th month MTP12 and 12th month MTP12, *p* = 0.621, 0.157, 0.097, 0.09, r = -0.115, -0.32, -0.37, -0.38 respectively).

**Table 3:** Correlations of MTP values. Bold values are significant data.

Correlations		1^st^ Month MTP	6^th^ Month MTP6	6^th^ Month MTP12	12^th^ Month MTP12
1^st^ Month MTP	r	1	0.765	0.78	0.608
	p	.	0	0	0.003
6^th^ Month MTP6	r	0.765	1	0.989	0.754
	p	0	.	0	0
6^th^ Month MTP12	r	0.78	0.989	1	0.779
	p	0	0	.	0
12^th^ Month MTP12	r	0.608	0.754	0.779	1
	p	0.003	0	0	.

There was no correlation between the MTP values at any time with the diameter of the ipsilateral cochlear nerve. However, the first month MTP, 6th month MTP6 and 12th month MTP12 correlated with the cross-sectional area of the ipsilateral cochlear nerve (*p* = 0.012, 0.034, 0.046 and r = 0.53, 0.46, 0.43 respectively) (Table 4). Although not significantly correlated BIS12 test results were better in patients with higher CSA (*p* = 0.066, r = 0.4).

**Table 4:** Correlations between MTP and BIS values and ipsilateral cochlear nerve diameter and cross-sectional area. Bold values denote significant data.

		Ipsilateral	
		Diamater	Area
1^st^ Month MTP	r	0.13	0.53
	p	0.55	0.012
6^th^ Month MTP6	r	0.13	0.46
	p	0.56	0.034
6^th^ Month MTP12	r	0.14	0.43
	p	0.54	0.052
12^th^ Month MTP12	r	0.11	0.43
	p	0.61	0.046
12^th^ Month BIS12	r	0.05	0.4
	p	0.81	0.066

When the MTP results were converted to fail and pass according to 70% success rate, 6th month MTP6 values were not significantly different from ipsilateral cochlear nerve diameter but were significantly different from ipsilateral cochlear nerve cross-sectional area (Table 5) (*p* = 0.32 and 0.012 respectively). In 12th month MTP12 test there was no significant difference between passed and failed patients and ipsilateral cochlear nerve diameter and cross-sectional area (Table 5) (*p* = 0.93 and 0.26 respectively).

**Table 5:** MTP test fail and pass and ipsilateral cochlear nerve diameter and cross-sectional area at 6th and 12th months. Bold values are significant data.

Cochlear nerve (ipsilateral)	6^th^ Month Fail		6^th^ Month Pass		*p*
	Mean ± SD	Med (Min − Max)	Mean ± SD	Med (Min − Max)	
Diameter	680.6 ± 108.3	667 (539.2 − 904.3)	720.8 ± 106.4	731 (505 − 867)	0.320
Area	0.014 ± 0.002	0.015 (0.012 − 0.016)	0.015 ± 0.001	0.016 (0.015 − 0.018)	0.012
	12^th^ Month Fail		12^th^ Month Pass		*p*
	Mean ± SD	Med (Min − Max)	Mean ± SD	Med (Min − Max)	
Diameter	702.0 ± 61.6	718 (601 − 764)	699.1 ± 114.2	693 (504 − 904)	0.93
Area	0.014 ± 0.001	0.015 (0.012 − 0.016)	0.015 ± 0.001	0.015 (0.012 − 0.018)	0.260

Mann-Whitney *U* test.

## Discussion

Cochlear implantation is an effective treatment method for the rehabilitation of profound sensorineural hearing loss. However, outcomes of cochlear implantation are highly variable and mostly depend on individual medical/surgical factors as well as speech-language perspectives.^3^ Among these factors, anatomic conditions such as inner ear malformations have major impact on cochlear implant success. It is well known that cochlear nerve hypoplasia or aplasia influence variable outcomes following implantation and preoperative detection of these situations is important.^27^, ^28^ Although not hypoplastic or aplastic, the size of the cochlear nerve may affect the postoperative rehabilitation process. For this reason, we conducted this investigation to determine the effect of slight differences of cochlear nerve size on postoperative implant success.

Prior to cochlear implant surgery comprehensive evaluation of the patients is mandatory to predict the outcomes. For the evaluation of inner ear structures, computed tomography and MRI of the temporal bone is mandatory. CT images provide very detailed information about the osseous structures of the inner ear, but its ability to identify nerves within the internal acoustic canal is limited.^29^ Whereas MRI can be very helpful to visualize nerves and brain structures in cochlear implant candidates. Patients with normal internal acoustic canal diameter in CT images can have absent cochlear nerves in MRI and clinicians should not rely only on CT findings about inner ear morphology.^29^ For these reasons morphology of the cochlear nerve should be analyzed on the MRI by millimetric measurements or by comparing the size to the size of the facial nerve.^30^

There are several studies that used radiologic evaluation of the size of the cochlear nerve in various patient groups and in normal hearing individuals. Lou et al. investigated the size of the cochlear nerve at 3 measurement points by 3-T MRI and analyzed the effect of age on cochlear nerve size in normal hearing children. They found that the largest value of the nerve was in the midpoint of IAC and the size did not change with age.^31^ Herman et al. measured the cross-sectional area of the cochlear nerve in normal hearing and postlingually deafened patients with MRI and concluded that parasagital CISS MRI was a reliable technique to measure the cochlear nerve. They found that CSA of the deaf patients was significantly smaller than in the normal hearing individuals.^32^ Russo et al. used high-resolution MRI to evaluate cochlear nerve size in children with sensorineural hearing loss and normal hearing and found that cochlear nerves were slightly hypoplastic in patients with profound hearing loss. They concluded that accurate measurements of the cochlear nerve could be done with high resolution MRI.^18^ Stjernholm and Muren used CT to determine cochlear nerve diameters and nerve size of the temporal bones and measured the nerve canal radiologically to compare to the measurements done on the casts of the same temporal bones. They found that the measurements on the casts were larger than the CT measurements. They concluded that the measurements on CT images might not pass exactly from the midpoint of the nerve and might not reflect the real measurements.^33^ We also used high resolution MRI to evaluate the size of the cochlear nerve. We included patients with normal- appearing and normal- sized cochlear nerves when the criteria implyied that cochlear nerves are slightly larger than facial nerves and this may be used in the determination of cochlear nerve abnormalities proposed by Miyasaka.^30^ The facial nerve has served as a valid reference for assessment of cochlear nerve in patients with hearing loss in previous studies.^34^

In this study the parasagittal reformatted images were used to visualize the cochlear nerve and measurements were done on these images. As the diameter measurements might not reflect the real size of the nerve, and as the cochlear nerves might not be circular and the measurement point might not pass from the midpoint, we also calculated the CSA. Our findings did not reveal any difference between the implanted and the contralateral side in our patient group. Also, there was no difference in the nerve diameter and CSA among gender and age. In pediatric population as development and growth continues, a concern about the change of cochlear nerve size with age arises. However, from the embryologic studies the vestibulocochlear nerve was showed to be fully developed at 6th embryologic life and did not change with physical development.^35^ Furthermore, there are multiple papers that investigated the size of the cochlear nerve in different age groups from newborn to elderly. Russo et al. in their study with normal hearing children found no difference in cochlear nerve diameter in different age groups.^18^ Lou et also showed no significant difference in the diameters of children’s CNs with age through more detailed grouping by providing imaging evidence.^31^ So, in this study we did not divide the patients according to age as the age does not influence the diameter of the cochlear nerve. Also, in this study our concern was not to investigate the effect of age on nerve size but to study whether the effect of minor differences of nerve size effect the hearing outcome independent of age factor.

It is also logical to think whether the implant brand may affect the hearing outcomes. In a study conducted by Bazon et al. two different speech coding strategies of different brands were compared and they found no significant difference in speech perception.^36^ Yet in another study Jang et al. worked on two different cochlear implant brands to investigate the postoperative performance and they found that there was no difference on open-set monosyllabic word, open-set disyllabic word and open-set sentence scores.^37^ So, in this study we did not discriminate between the implant brands and we believe that postoperative performance of each brand is similar.

EARS assessment tool evaluates auditory perception development following cochlear implantation, and provides information for device fitting and provides an instrument for the long-term assessment of children with cochlear implants.^38^ The EARS tool was used in many previous studies to evaluate auditory perception development in the same patient group.^39^ The tool was also used to compare patients with different age groups^40^ or patients with the same age but different groups with various etiologic factors for hearing loss.^41^ Although different age groups and different etiologic factors on hearing loss demonstrate different improvement charts, in general following implantation and rehabilitation, auditory perception improves over time and this improvement occurs steadily over 6 month or 1-year intervals.^38^ Also the studies demonstrated that the earlier the implantation the better the auditory responses.^39^ In this study we evaluated our patients with EARS assessment tool. The assessment was done on 1st month, 6th month and 12th month with different tools and we detected that MTP test responses increased steadily over time, and the patients with high score on 1st month MTP demonstrated high scores on the following tests. Also, the patients that were implanted at a younger age achieved higher scores on the entire tests although the results were not statistically significant.

Although there are many radiology reports on cochlear nerve size, the knowledge on the implication of cochlear nerve size on implant success in prelingually deaf children is limited. Yamazaki et al. classified patients according to the ratio between facial nerve and cochlear nerve by MRI and found that patients with cochlear nerves larger than facial nerves performed better following cochlear implantation.^42^ Morita et al. evaluated the relationship between cochlear implant outcome and the diameter of the cochlear nerve on MRI in 20 prelingually deaf children and found that there was no correlation between the maximal diameters of the nerves and age, ECAP values and IT-MAIS scores. They concluded that identification of the cochlear nerve on MRI was enough to expect better effects regardless of the diameters.^14^ Kim et al. investigated the correlation of cochlear nerve size and auditory performance after cochlear implantation in postlingual patients. They assessed 68 patients and measured the cochlear nerve diameter and CSA on MR images. They found that CSA was negatively associated with duration and the degree of hearing loss. They also detected that CSA was positively correlated with auditory performance.^21^ In our study we included prelingually deaf children and since measuring only the diameter might not reflect the real size of the nerve, we calculated cochlear nerve diameter as well as CSA of the nerve. In our group none of the patients had hypoplastic or aplastic cochlear nerves (the cochlear nerves were larger than the facial nerves). As expected, and demonstrated by two previous studies, the diameter of the nerves was not significantly correlated to postoperative audiologic performance. This may be because the nerve may not be circular in shape and the measurements may not reflect the real size. However, the cross-sectional area of the cochlear nerves was significantly correlated to postoperative auditory perception. In patients with higher CSA had better MTP scores on 1st, 6th and 12th month assessments. Nadol and Xu measured the cochlear nerves in cadavers and found that in cadavers with small nerves fewer residual spiral ganglion cells were present.^43^ The cross-sectional area may correlate with residual spiral ganglion cells that improves cochlear implant outcomes.^32^ We believe that CSA of the cochlear nerve gives better information on the morphology of the nerve since irregular shape of the nerve may underscore the diameter. Also, the CSA may reflect the density of the spiral ganglion cells that explains better outcomes. We think that although normal- appearing slight differences on CSA of the cochlear nerve may affect performance, measuring the size of the cochlear nerve on parasagittal MR images may provide beneficial information on postoperative rehabilitation process.

## Conclusion

Among many factors influencing cochlear implant hearing outcomes, the size of cochlear nerve may be the most important factor although normal- sized minor changes in nerve diameter may yield different hearing results. Measuring the CSA of the normal -appearing cochlear nerve may give important prognostic knowledge on cochlear implant outcomes. Our study demonstrated that in patients with larger CSA, the postoperative auditory performance was better and faster.

## Conflicts of interest

The authors declare no conflicts of interest.
